# *In vivo* imaging of CD8^+^ T cells in metastatic cancer patients: first clinical experience with simultaneous [^89^Zr]Zr-Df-IAB22M2C PET/MRI

**DOI:** 10.7150/thno.79976

**Published:** 2023-04-17

**Authors:** Johannes Schwenck, Dominik Sonanini, Dominik Seyfried, Walter Ehrlichmann, Gabriele Kienzle, Gerald Reischl, Pascal Krezer, Ian Wilson, Ron Korn, Irene Gonzalez-Menendez, Leticia Quintanilla-Martinez, Ferdinand Seith, Andrea Forschner, Thomas Eigentler, Lars Zender, Martin Röcken, Bernd J Pichler, Lukas Flatz, Manfred Kneilling, Christian la Fougere

**Affiliations:** 1 Department of Nuclear Medicine and Clinical Molecular Imaging, Eberhard Karls University, Tübingen, Germany.; 2 Werner Siemens Imaging Center, Department of Preclinical Imaging and Radiopharmacy, Eberhard Karls University, Tübingen, Germany.; 3 Cluster of Excellence iFIT (EXC 2180) "Image-Guided and Functionally Instructed Tumor Therapies", Eberhard Karls University, Tübingen, Germany.; 4 Department of Medical Oncology and Pneumology (Internal Medicine VIII), Eberhard Karls University, Tübingen, Germany.; 5 ImaginAb, Inc., Inglewood, California.; 6 Institute of Pathology and Neuropathology, Comprehensive Cancer Center, Eberhard Karls University, Tübingen, Germany.; 7 Department of Diagnostic and Interventional Radiology, Eberhard Karls University, Tübingen, Germany.; 8 Department of Dermatology, Eberhard Karls University, 72076 Tübingen, Germany.; 9 Charité - Universitätsmedizin Berlin, corporate member of Freie Universität Berlin and Humboldt-Universität zu Berlin, Department of Dermatology, Venereology and Allergology, Luisenstrasse 2, Berlin, 10177, Germany.; 10 German Cancer Consortium (DKTK), German Cancer Research Center (DKFZ) Partner Site Tübingen, Tübingen, Germany.

## Abstract

**Aim/Introduction:** Despite the spectacular success of immune checkpoint inhibitor therapy (ICT) in patients with metastatic cancer, only a limited proportion of patients benefit from ICT. CD8^+^ cytotoxic T cells are important gatekeepers for the therapeutic response to ICT and are able to recognize MHC class I-dependent tumor antigens and destroy tumor cells. The radiolabeled minibody [^89^Zr]Zr-Df-IAB22M2C has a high affinity for human CD8^+^ T cells and was successfully tested in a phase I study. Here, we aimed to gain the first clinical PET/MRI experience with the noninvasive assessment of the CD8^+^ T-cell distribution in cancer patients by* in vivo* [^89^Zr]Zr-Df-IAB22M2C with a distinct focus of identifying potential signatures of successful ICT.

**Material and Methods:** We investigated 8 patients with metastasized cancers undergoing ICT. Radiolabeling of Df-IAB22M2C with Zr-89 was performed according to Good Manufacturing Practice. Multiparametric PET/MRI was acquired 24 h after injection of 74.2±17.9 MBq [^89^Zr]Zr-Df-IAB22M2C. We analyzed [^89^Zr]Zr-Df-IAB22M2C uptake within the metastases and within primary and secondary lymphatic organs.

**Results:** [^89^Zr]Zr-Df-IAB22M2C injection was tolerated well without noticeable side effects. The CD8 PET/MRI data acquisitions 24 hours post-administration of [^89^Zr]Zr-Df-IAB22M2C revealed good image quality with a relatively low background signal due to only low unspecific tissue uptake and marginal blood pool retention. Only two metastatic lesions showed markedly increased tracer uptake in our cohort of patients. Furthermore, we observed high interpatient variability in [^89^Zr]Zr-Df-IAB22M2C uptake within the primary and secondary lymphoid organs. Four out of five ICT patients exhibited rather high [^89^Zr]Zr-Df-IAB22M2C uptake in the bone marrow. Two of these four patients as well as two other patients yielded pronounced [^89^Zr]Zr-Df-IAB22M2C uptake within nonmetastatic lymph nodes. Interestingly, cancer progression in ICT patients was associated with a relatively low [^89^Zr]Zr-Df-IAB22M2C uptake in the spleen compared to the liver in 4 out of the 6 patients. Lymph nodes with enhanced [^89^Zr]Zr-Df-IAB22M2C uptake revealed significantly reduced apparent diffusion coefficient (ADC) values in diffusion weighted MRI.

**Conclusion:** Our first clinical experiences revealed the feasibility of [^89^Zr]Zr-Df-IAB22M2C PET/MRI in assessing potential immune-related changes in metastases and primary and secondary lymphatic organs. According to our results, we hypothesize that alterations in [^89^Zr]Zr-Df-IAB22M2C uptake in primary and secondary lymphoid organs might be associated with the response to ICT.

## Introduction

The introduction of immune checkpoint inhibitor therapy (ICT) in 2011 for metastatic melanoma represented a milestone in the treatment of advanced cancer. Previously, the prognosis of those patients was poor, with a 5-year survival rate of about 5% and an overall survival (OS) of approximately seven to eight months 1. The use of monoclonal antibodies directed against the immune checkpoints CTLA4 (cytotoxic T-lymphocyte antigen 4, ipilimumab) and anti-PD-1 (programmed cell death protein 1, nivolumab and pembrolizumab) significantly improved both the recurrence-free survival and OS of cancer patients. In individual cases, patients with relapse-free long-term survival have even been reported 1. PD-1 and CTLA4 are inhibitory receptors expressed particularly on T cells. The antibody-mediated blockade of these inhibitory receptors reinvigorates the T-cell response, resulting in improved tumor control 2. ICT is now a mainstay of systemic cancer therapy and has been firmly established in the relevant guidelines 3.

However, despite decreasing mortality rates in cancer patients treated with ICT, only a limited number of patients benefit from this expensive treatment approach. Only approximately 40% of melanoma patients respond to anti-PD-1 antibody monotherapy, while combining anti-CTLA4 and anti-PD-1 antibodies increases the response rate up to 60% 4, 5. Regardless of treatment success, patients frequently develop immune-related adverse events (irAEs) that can lead to significant comorbidities in almost every organ, including treatment-related death 6, 7. To date, no reliable predictive biomarker for ICT is available.

Activated effector CD8^+^ T cells are an important prerequisite for successful ICT 8. The spatial distribution of CD8^+^ cytotoxic T cells seems to play an important role both microscopically in the tumor 9 and macroscopically in the entire organism 10, 11. A preexisting infiltration of CD8^+^ T cells was shown to be crucial for effective ICT 12; therefore, tumor-infiltrating CD8^+^ T cells are considered to be a potential surrogate marker for the response to ICT 13.

CD8 is a cell surface protein that functions as a coreceptor in antigen recognition on major histocompatibility complex class I (MHC I) molecules and is expressed on cytotoxic T cells but also on some natural killer (NK) cells, natural killer T cells (NKT) and certain populations of dendritic cells 14, 15. CD8^+^ T cells are found in most adult lymphoid tissues as well as in the blood where they perform their physiological task, e.g., in the defense against viral infections 16, 17.

Nevertheless, in recent years, it has been discovered that not all subsets of CD8^+^ T cells are capable of effectively attacking tumor cells. In particular, terminally exhausted T cells lose their effector functions and express high levels of PD-1. In contrast, “stem-like” progenitor exhausted CD8^+^ T cells possess the ability to proliferate and are considered crucial for persistent T-cell responses and successful ICT 18-20. These cells are characterized by the expression of T-cell Factor 1 (TCF1) and intermediate levels of PD-1 19. The zirconium-89-desferrioxamine (^89^Zr-DFO)-labeled CD8-directed scFv-CH3 antibody fragment (minibody), [^89^Zr]Zr-Df-IAB22M2C, is characterized by a high affinity for human CD8^+^ T cells, enabling *in vivo* T-cell tracking. In the dose escalation phase of the first phase I study, six cancer patients were injected with different [^89^Zr]Zr-Df-IAB22M2C doses (0.2 mg-10 mg; 111 MBq) 21. A dose-dependent tracer kinetic with high accumulations in lymphoid tissues such as spleen, bone marrow, and lymph nodes was observed by PET imaging between 2 and 148 h post-injection (*p.i.*). Of note, increased tracer uptake in metastases was found in only two out of six patients 21. In a recent publication including the dose expansion phase of the phase I trial with 9 additional patients, an association between tumor uptake and response to immunotherapy was described in two melanoma patients under anti-PD-1 therapy 22.

Nevertheless, the meaning of the whole-body distribution of CD8 expression patterns is still unexplored and inconclusive, especially with regard to successful cancer immune responses.

Considering the enormous molecular biology efforts to characterize the fate of CD8^+^ T-cells into very distinct cellular subsets, it is obvious that a simple assessment of CD8 expression, e.g., in the tumor microenvironment, might not be capable of assessing effective immune responses due to the different functionality of the CD8^+^ T-cell subsets. A plethora of markers have been proposed in recent years to define CD8^+^ T-cell subsets, such as Tox, T-bet, and Tcf1 23. Clinical *in vivo* imaging cannot keep up with the multitude of molecular markers provided by molecular biology methods, such as single-cell RNA sequencing, for the characterization of functional CD8^+^ T-cell subsets due to technical and practical reasons. However, a unique feature of whole-body [^89^Zr]Zr-Df-IAB22M2C PET/MRI compared to other diagnostic methods is the capability of assessing the spatial distribution of CD8 expression *in vivo* in a quantitative manner. Therefore, different image analysis strategies, focusing on the interplay of the different lymphatic tissues and the tumor, need to be explored to extract the relevant information to evaluate an efficient cancer immune response.

Strong evidence from preclinical and clinical findings emphasize the essential role of systemic immunity including peripheral T cells in the blood 24, lymph nodes 25, and spleen for effective cancer immunity 26. ICT demonstrably altered T cell activity and motility, increased the number of antitumor CD8^+^ T cells in tumor-draining lymph nodes and thus enhanced the antitumor response 27.

Specific CD8^+^ T cells expand in the draining lymph node after contact with the tumor associated antigen which is not only essential for successful ICT but also forms part of physiological immune reactions e.g. in response to viruses 28-30. Nevertheless, also resting, steady-state lymph nodes contain CD8^+^ T cells although homing mechanisms are different to inflamed lymph nodes 31.

CD8^+^ T cells in the spleen are highly involved in mounting systemic immune responses 32, 33, but are also important in the immune tolerance of tumors 34. Additionally, the rare cases of autosplenectomy associated with ICT suggest a strong involvement of the spleen 35, 36. Besides T cells, littoral cells, which are lining the sinusoidal structures of the red pulp in the spleen, express high levels of CD8, although the heritage or the functional significance of the CD8 expression is unknown in these cells 37, 38.

The bone marrow is the major primary lymphoid organ in adults, but nevertheless the bone marrow also functions as important secondary lymphoid organ including CD8^+^ T cell priming 39. A high abundance of naïve CD8^+^ T cells as well as effector and memory CD8^+^ T cells, have been described upon immune responses against cancer 40, 41 and homeostatic conditions 42-44. Evidence from hematological malignancies confirmed the involvement of bone marrow CD8^+^ T cells in ICT response 45-47.

Furthermore, several studies revealed that distinct functional CD8^+^ T-cell subsets are located in different tissue compartments 19. Progenitor exhausted T-cells in the lymphoid tissue are considered the main target of successful PD-1 therapy 48, 49. This is in line with findings emphasizing the essential role of tumor draining lymph nodes in ICT 29. In contrast, the interaction of terminally exhausted CD8^+^ T cells with M2 macrophages in tumor tissue was associated with a worse prognosis, e.g., in renal cell cancer 50.

Here, we report on CD8^+^ T-cell whole-body tracking in metastatic cancer patients with simultaneous [^89^Zr]Zr-Df-IAB22M2C PET/MRI. In contrast to previous publications, we mainly focused on holistic patient-specific differences in CD8^+^ T-cell distribution patterns within the primary and secondary lymphatic organs. In addition, we determined the apparent diffusion coefficient (ADC) values in eligible lymph nodes with differential [^89^Zr]Zr-Df-IAB22M2C uptake. These approaches might have the potential to identify signatures of successful ICT that could be used for treatment stratification in subsequent studies and finally in routine clinical care.

## Material and Methods

Following the stipulations of the German medicinal products act (*“Arzneimittelgesetz”*; AMG) §13(2b), PET/MRI scans with [^89^Zr]Zr-Df-IAB22M2C were performed in 8 patients with metastasized cancers after they provided written informed consent (5 with cutaneous melanoma, 1 with choroidal melanoma, 1 with NSCLC and 1 with sarcoma). PET/MRI was preferred to PET/CT to reduce radiation exposure. Three patients were eligible for ICT, and the referring physicians requested [^89^Zr]Zr-Df-IAB22M2C PET imaging to aid in the clinical decision between ICT or therapeutic alternatives. Five patients were undergoing ICT, and clinical decisions for prolongation, escalation or change of treatment needed to be addressed by the referring physicians. Six out of eight patients presented with progressive disease in the follow-up after the [^89^Zr]Zr-Df-IAB22M2C PET/MRI and were considered nonresponders. Patients 3 and 4 showed long-term remission without detectable metastasis in the routine follow-up for more than one year after [^89^Zr]Zr-Df-IAB22M2C PET/MRI (responders).

### Radiolabeling

[^18^F]FDG was manufactured in-house under a marketing license.

Patient individual preparations of [^89^Zr]Zr-Df-IAB22M2C were performed following a modified procedure of Pandit-Taskar et al. 21 in a GMP environment. The precursor Df-IAB22M2C (80 kDa) was provided by ImaginAb Inc. (Inglewood, CA, USA), and Zr-89 was manufactured by BV Cyclotron VU (Amsterdam, The Netherlands) and purchased from PerkinElmer (Rodgau, Germany). All other reagents, in the highest quality available, were from common suppliers. Briefly, in a sterile, pyrogen-free tube, the acidic solution of [^89^Zr]zirconium-oxalate was neutralized with 2 M sodium carbonate solution and buffered with 0.5 M ammonium acetate solution to a pH value of 6.5-7.5, and then the Df-IAB22M2C precursor solution (protein concentration 2.7 mg/mL) was added. The ^89^Zr activity and precursor amounts for a patient dose were calculated from the first test runs (taking into account losses during preparation) to obtain the values described in the Materials and Methods Section titled “*CD8 PET/MRI*”. After reaction for 40 min at ambient temperature, 10 µL of DTPA solution (20%) was added to react with residual Zr-89 for 10 min. The efficiency of labeling was checked by TLC chromatography and autoradiography (≥ 94%, n = 8). Purification was performed using size-exclusion chromatography (disposable PD10 column, product code: 17085101, GE Healthcare). After elution of the product with the final formulation matrix (water for injection, 20 mM histidine, 5% (v/v) sucrose, 50 mM NaCl, 0.2 M L-arginine, pH 6.5), the product was sterile filtered, and samples were taken for quality control. Every product batch was tested for the following parameters using validated methods (specifications in brackets): appearance (clear, colorless, free of visible particles), pH (4.0-8.0), radiochemical purity tlc (≥ 90%), radiochemical purity size-exclusion HPLC (≥ 80%), radionuclidic purity, gamma spectrometry (≥ 99.9%), endotoxins (< 17.5 EU/mL, volume of patient dose ≤ 10 mL), immunoreactivity, bead assay (≥ 70%), and sterility test (no growth). Every prepared batch met the specifications (n = 8).

### CD8 PET/MRI

Multiparametric CD8 PET/MRI was performed 24 h after injection of 74.2 ± 17.9 MBq [^89^Zr]Zr-Df-IAB22M2C (1.1-1.8 mg Df-IAB22M2C) on a fully integrated 3T PET/MR system (Biograph mMR Siemens Healthineers, Erlangen, Germany). Previously published studies by Pandit-Taskar et al. and Farwell et al. reported a maximum tracer uptake 24-48 h p.i. 21, 22, and for patient convenience, we opted for PET/MRI data acquisition at 24 h p.i. To minimize radiation exposure to patients, we reduced the amount of tracer activity administered compared with the previously reported value available in the literature data (111 MBq 21, 22; see Table S1).

PET data (4 min per bed position, four bed positions, cranial vertex to the thighs) were reconstructed using the vendor's software with the 3D-OSEM algorithm, 21 subsets, 2 iterations, 256 × 256 matrix size (voxel size: 2.8 × 2.8 × 2.0 mm^3^) and a 4 mm Gaussian filter. A 3D T1-weighted spoiled gradient-echo sequence with Dixon-based fat-water separation in end-expiratory breath-hold was acquired to create an attenuation map. All attenuation maps were checked carefully for erroneous tissue identification. Different MRI sequences were acquired depending on clinical questions, which included, inter alia, a fat saturated postcontrast T1 volumetric interpolated breath-hold examination (VIBE) acquired in axial view with multiple breath-holds for anatomic correlation. Whole-body diffusion weighted imaging (DWI) was performed using a 192 × 168 image matrix, 5 mm slice thickness, response time 3500ms, echo time 52ms, spectral attenuated inversion recovery (SPAIR) fat suppression, pixel size 1.6 x 1.6mm, image matrix 192 x 168 and b values of 50 and 800 s/mm^2^. The ADC maps were created using the proprietary software from the vendor.

### Image analysis

CD8 PET/MRI data were reviewed by two board certified nuclear medicine specialists and discussed with the referring clinicians. For semiquantitative analysis, representative regions of interest (ROIs) with 50% isocontour were defined by using a dedicated software package (Syngo.via, Siemens Healthineers, Erlangen and Affinity Viewer, Hermes Medical Solutions, Stockholm, Sweden). Lymph nodes and lesions with discernable tracer uptake compared to the background on fused PET/MR images were rated as positive for CD8 expression. Lesions were defined as malignant in concordance with the clinical reports of the available previous and subsequent routine imaging studies, including [^18^F]FDG PET/CT and CT scans. As the immune infiltrate in the lymphatic organs as well as the malignant tissue are subject to highly dynamic temporal changes we only considered [^18^F]FDG PET scans in a narrow timeframe of 6 weeks within the [^89^Zr]Zr-Df-IAB22M2C PET (see Table S2). To select representative lymph nodes for the analysis of the ADC, we chose the lymph nodes with the highest uptake in the region which had a sufficient diameter for a reliable assessment of the ADC. Lymph nodes without distinguished uptake compared to the surrounding tissue were considered as negative. In a similar fashion to the CD8 positive lymph nodes, we chose CD8 negative lymph nodes with comparable diameter.

### Histology and immunohistochemistry

The morphological and immunohistochemical features were analyzed on formalin-fixed and paraffin-embedded tissue sections. Samples of Patient 1 (9 weeks after the [^89^Zr]Zr-Df-IAB22M2C PET/MRI) and Patient 3 (6 weeks after [^89^Zr]Zr-Df-IAB22M2C PET/MRI) were stained with hematoxylin and eosin (H&E). Additionally, CD8 immunohistochemistry was performed on an automated immunostainer (Ventana Medical Systems, Tucson, AZ) according to the manufacturer's protocol using a CD8 antibody (clone C8/144B; Dako, Glostrup, Denmark). Appropriate positive and negative controls were used to confirm the adequacy of the staining. All samples were scanned with the Ventana DP200 (Roche, Basel, Switzerland) and processed with the Image Viewer MFC Application. Final image preparation was performed with Adobe Photoshop CS6.

### Data Availability

The data that support the findings of this study are available from the corresponding authors upon reasonable request.

## Results

The injection of 74.2±17.9 MBq [^89^Zr]Zr-Df-IAB22M2C was well tolerated in all patients without significant clinical side effects. In particular, we observed no allergic reactions, cutaneous erythema at the injection site, nausea, vomiting or circulatory problems from tracer injection until the end of the PET/MRI measurement. The PET/MRI acquisitions 24 h *p.i.* revealed good image quality. We noted a marginal background signal due to a minor retention in the blood pool and some marginal nonspecific tissue uptake.

In total, eight patients with metastatic cancer (cutaneous melanoma, n = 5; choroidal melanoma, n = 1; NSCLC, n = 1; sarcoma, n = 1) were assessed by means of PET/MRI before (n = 3) or during (n = 5) ICT (melanoma, n = 4; sarcoma, n = 1). Patients underwent anti CTLA-4 (ipilimumab, n = 1), anti PD-1 (nivolumab or pembrolizumab, n = 3) or combined checkpoint inhibition with ipilimumab and nivolumab (n = 1) (Table 1). Detailed findings of [^89^Zr]Zr-Df-IAB22M2C PET/MRI are shown in Table 1. Only two melanoma patients (Patient 1 and Patient 7) exhibited increased [^89^Zr]Zr-Df-IAB22M2C uptake in metastasis compared to the background; all other metastases did not reveal any relevant tracer uptake.

**Figure 1:**
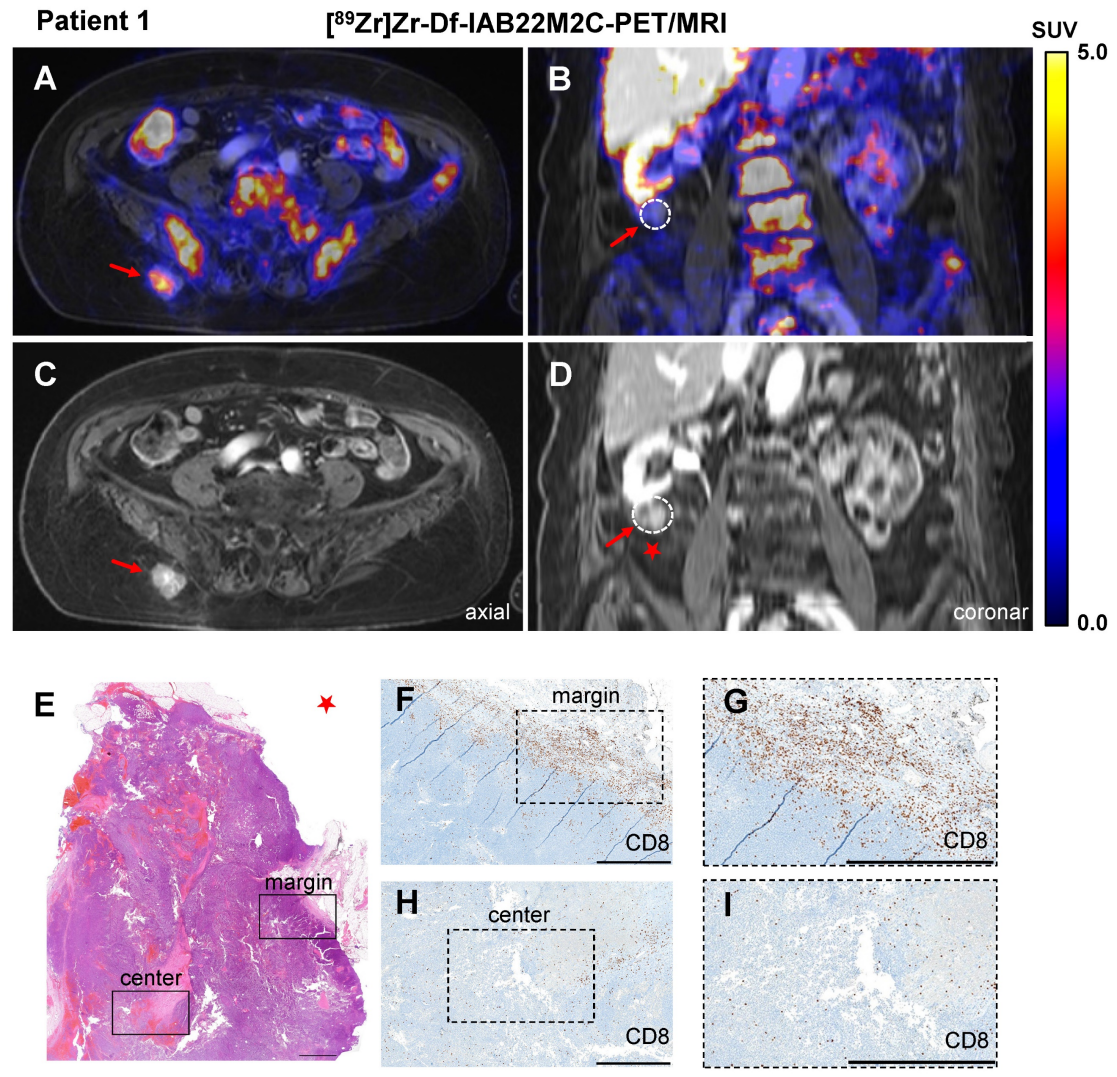
** A/C:** Axial fused [^89^Zr]Zr-Df-IAB22M2C PET/MRI and MRI of a metastasis with intense tracer uptake in the right gluteal subcutaneous tissue of Patient 1 after three cycles of ipilimumab until 6 weeks before the scan. **B/D:** Coronal fused [^89^Zr]Zr-Df-IAB22M2C PET/MRI and MRI of a pararenal metastasis of Patient 1 without relevant tracer uptake after ipilimumab treatment. **E-I:** The pararenal metastasis of Patient 1 (red star) was resected. Histopathological analysis revealed monotonous cell proliferation in the metastasis **(E)**. Immunohistochemistry revealed some areas with dense infiltrates of CD8^+^ T cells in the margin of the lesion **(F)**; magnification** (G)**, mild infiltration of CD8^+^ T cells in the center of the metastasis (focally moderate around the necrotic areas) was observed **(H)**; magnification** (I).** Scale bar: 1 mm for both H&E and CD8 immunohistochemistry images.

In Patient 1, CD8 PET/MRI was performed after three cycles of ipilimumab treatment, which was discontinued 6 weeks prior to the PET/MR scan due to recurrence of rheumatoid arthritis. CD8 PET/MRI revealed intense [^89^Zr]Zr-Df-IAB22M2C uptake in a progressive subcutaneous gluteal metastasis (SUVmean 3.4; Figure 1A-B). Notably, a second progressive pararenal metastasis exhibited no relevant [^89^Zr]Zr-Df-IAB22M2C uptake (Figure 1C-D). Two other soft tissue lesions in the right thigh and presternal region were stable for a period of at least 3 months and did not show any relevant [^89^Zr]Zr-Df-IAB22M2C uptake.

Histopathological evaluation of the pararenal metastasis after surgical resection revealed monotonous cell proliferation of the melanoma metastasis (Figure 1E). CD8 immunohistochemistry showed dense CD8^+^ T-cell infiltrates in the periphery of the lesion (Figure 1F-G), while a mild infiltration of CD8^+^ T cells was found within the tumor (focally moderate around the necrotic areas; Figure 1H-I). As a consequence of progressive disease, planned resection of the gluteal metastasis was not performed.

**Figure 2:**
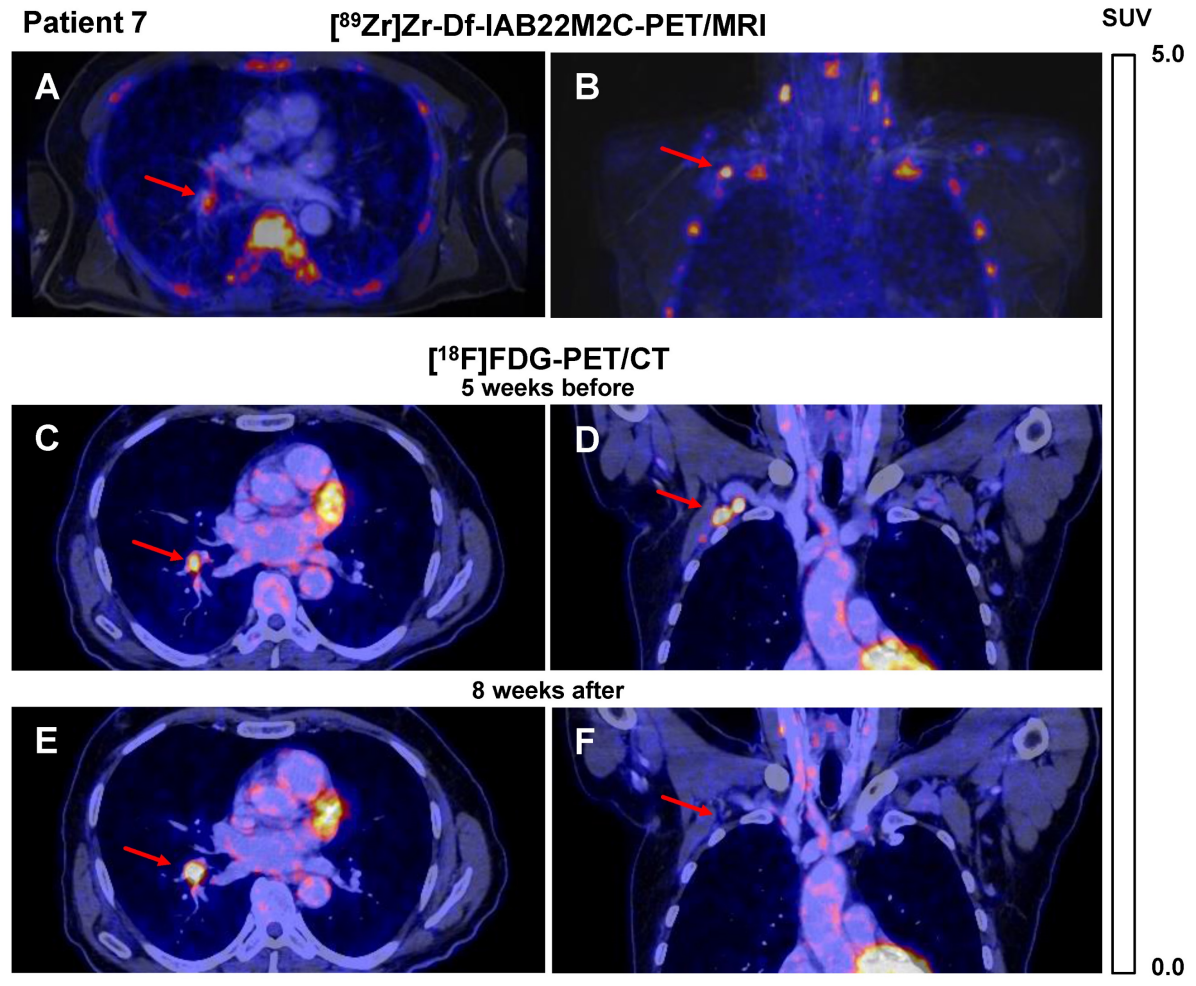
** A/B:** Axial **(A)** and coronal **(B)** fused [^89^Zr]Zr-Df-IAB22M2C PET/MRI of Patient 7 after 4 cycles of combined ICT using ipilimumab and nivolumab until 6 weeks before the scan indicated only faint tracer uptake in a hilar lymph node metastasis **(A)**, intense tracer uptake was found in an axillary lymph node metastasis **(B)**. **C/D:** Axial **(C)** and coronal **(D)** fused [^18^F]FDG PET/CT images of Patient 7 five weeks before [^89^Zr]Zr-Df-IAB22M2C PET/MRI indicated an intense glucose uptake of both the axillary and the hilar lymph node metastasis; these were considered to be viable tumor tissue. **E/F:** Eight weeks after [^89^Zr]Zr-Df-IAB22M2C PET/MRI, the follow-up [^18^F]FDG PET/CT of Patient 7 revealed a decrease in axillary lymph node metastasis during ICT **(F)**, while hilar lymph node metastasis **(E)** increased in size and [^18^F]FDG uptake during ICT monotherapy with nivolumab.

A similar [^89^Zr]Zr-Df-IAB22M2C uptake pattern was observed in Patient 7 after four cycles of combined ICT until 6 weeks before the [^89^Zr]Zr-Df-IAB22M2C PET/MRI. Different lymph nodes, which were described as suspicious for metastases in the previous routine [^18^F]FDG PET/CT performed five weeks earlier (e.g., the hilar lymph node metastasis), showed in this CD8 PET/MRI scan only weak [^89^Zr]Zr-Df-IAB22M2C uptake (SUVmean 2.8; Figure 2A), whereas axillary lymph node metastasis revealed intense [^89^Zr]Zr-Df-IAB22M2C uptake (SUVmean 6.6; Figure 2B). The corresponding [^18^F]FDG PET/CT indicated intense glucose metabolism in both metastases (Figure 2C-D). Interestingly, follow-up [^18^F]FDG PET/CT 8 weeks after [^89^Zr]Zr-Df-IAB22M2C PET/MRI revealed regression of the CD8 PET/MRI-positive axillary lymph node metastasis, while the CD8 PET/MRI-negative hilar lymph node metastasis increased in volume and [^18^F]FDG uptake (Figure 2E-F).

The quantification of the [^89^Zr]Zr-Df-IAB22M2C uptake of the metastases (subcutaneous; suspicious hilar and axillary lymph node) in the patients mentioned above revealed quite heterogeneous intra- and interindividual uptake patterns. Furthermore, background correction to the blood pool (metastasis to blood ratio) or secondary lymphatic organs (lymph nodes to blood or spleen to blood ratio) did not provide additional insights or enable a better understanding of CD8 distribution patterns (Figure S1A).

Patient 3 presented with a brain metastasis that was irradiated nine months before the [^89^Zr]Zr-Df-IAB22M2C PET/MRI and revealed no tracer uptake above background levels (Figure S2A). The histopathological analysis of the brain metastasis six weeks after PET/MRI revealed white and gray matter interspersed with gemistocytic astrocytes, together with necrotic tissue without viable malignant cells, consistent with postradiotherapy status (Figure S2). CD8 immunohistochemistry showed CD8^+^ T cells perivascularly near the necrotic areas, while only a few CD8^+^ T cells were identified in the brain parenchyma and necrotic areas (Figure S2C). In the left pararenal gland, where a residual metastasis was located (constant over years), we observed a relatively low [^89^Zr]Zr-Df-IAB22M2C uptake (SUVmean 4.5).

**Figure 3:**
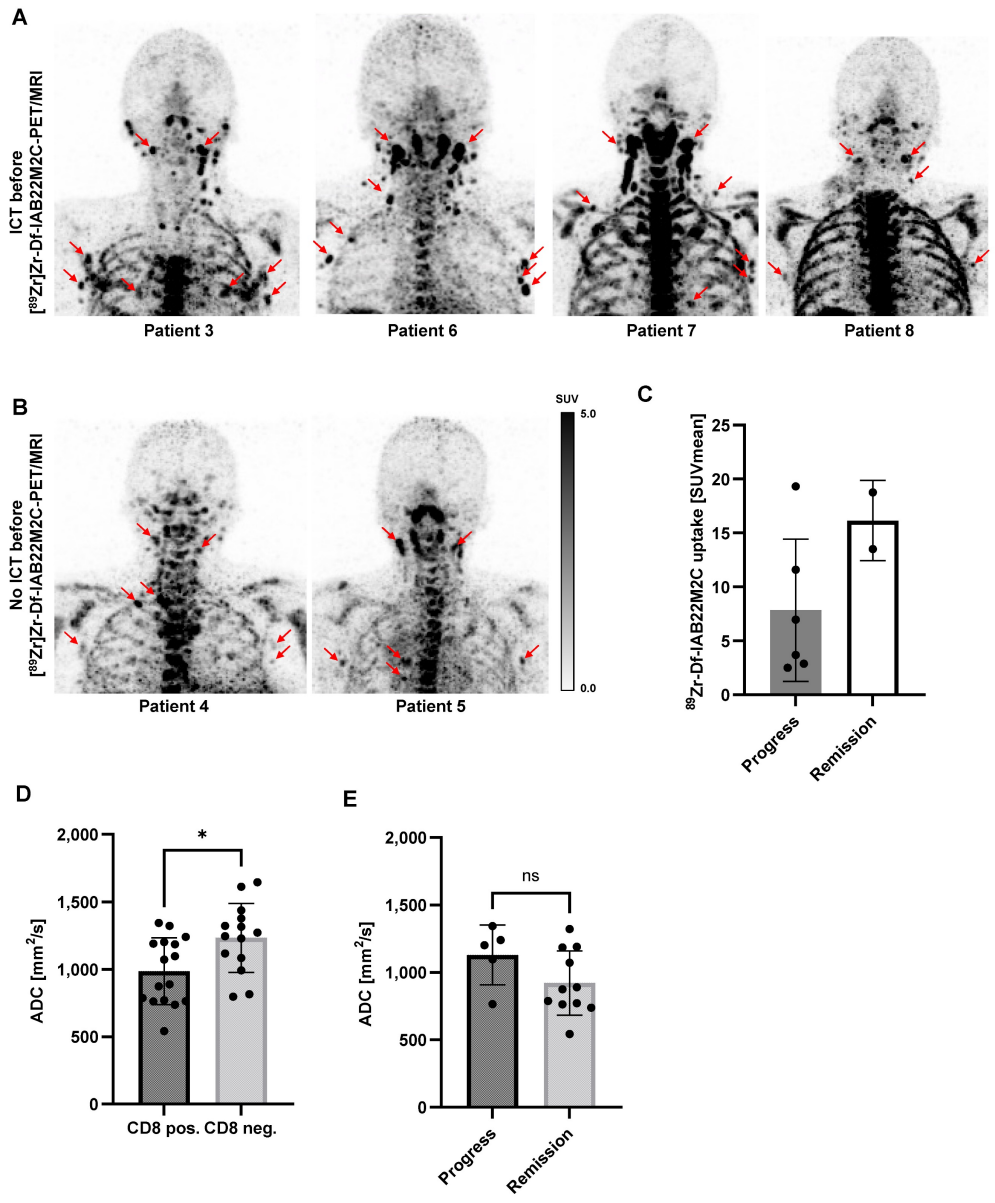
** A:** In Patients 3, 6, 7 and 8 with ICT pretreatment, multiple lymph nodes with intense [^89^Zr]Zr-Df-IAB22M2C uptake located predominantly in the cervical and thoracic regions were identified. Considering the previous routine imaging and the clinical history of the patients, these lymph nodes appeared to be associated with inflammatory processes rather than metastases.** B:** In Patients 4 and 5 without ICT pretreatment, intense [^89^Zr]Zr-Df-IAB22M2C uptake in the lymph nodes was evident. These lymph nodes have been considered nonmalignant according to previous routine imaging and clinical history.** C:** The lymph nodes with the highest [^89^Zr]Zr-Df-IAB22M2C uptake in the respective patients indicated a trend toward higher uptake values in the patients with remission. **D:** The apparent diffusion coefficient (ADC) was determined in up to three representative lymph nodes per patient with enhanced (CD8 pos.) or low [^89^Zr]Zr-Df-IAB22M2C uptake (CD8 neg.). The CD8 pos. lymph nodes revealed significantly reduced ADC values. **E:** CD8 positive lymph nodes of ICT-responsive patients revealed slightly higher ADC values than CD8 positive lymph nodes of nonresponsive patients.

Multiple lymph nodes (predominantly in the cervical and thoracic regions) with intense [^89^Zr]Zr-Df-IAB22M2C uptake, most likely associated with inflammatory processes, were found in 6 out of 8 patients. Metastatic spread in these lymph nodes appeared implausible when considering previous clinical CT or [^18^F]FDG PET/CT imaging and the clinical history of the patients. These considerably inflamed lymph nodes with a very high [^89^Zr]Zr-Df-IAB22M2C uptake were observed in four out of five patients with previous ICT treatment (Figure 3A). However, two out of three patients without ICT (Patient 4 and Patient 5) exhibited an identical phenomenon (Figure 3B). The quantification of the lymph nodes with the highest [^89^Zr]Zr-Df-IAB22M2C uptake in the respective patients indicated a trend toward increased uptake values in the patients with long-term remission upon ICT (Figure 3C and Table 1).

Next, we asked whether differences in diffusion were associated with increased [^89^Zr]Zr-Df-IAB22M2C uptake. Diffusion-weighted MRI (DWI) of eligible lymph nodes with enhanced or low [^89^Zr]Zr-Df-IAB22M2C uptake revealed significantly reduced apparent diffusion coefficient (ADC) values in lymph nodes with enhanced [^89^Zr]Zr-Df-IAB22M2C uptake (Figure 3D). Additionally, we observed a tendency towards higher ADC values in the CD8 positive lymph nodes of ICT-responsive patients compared to nonresponsive patients (Figure 3E).

**Figure 4:**
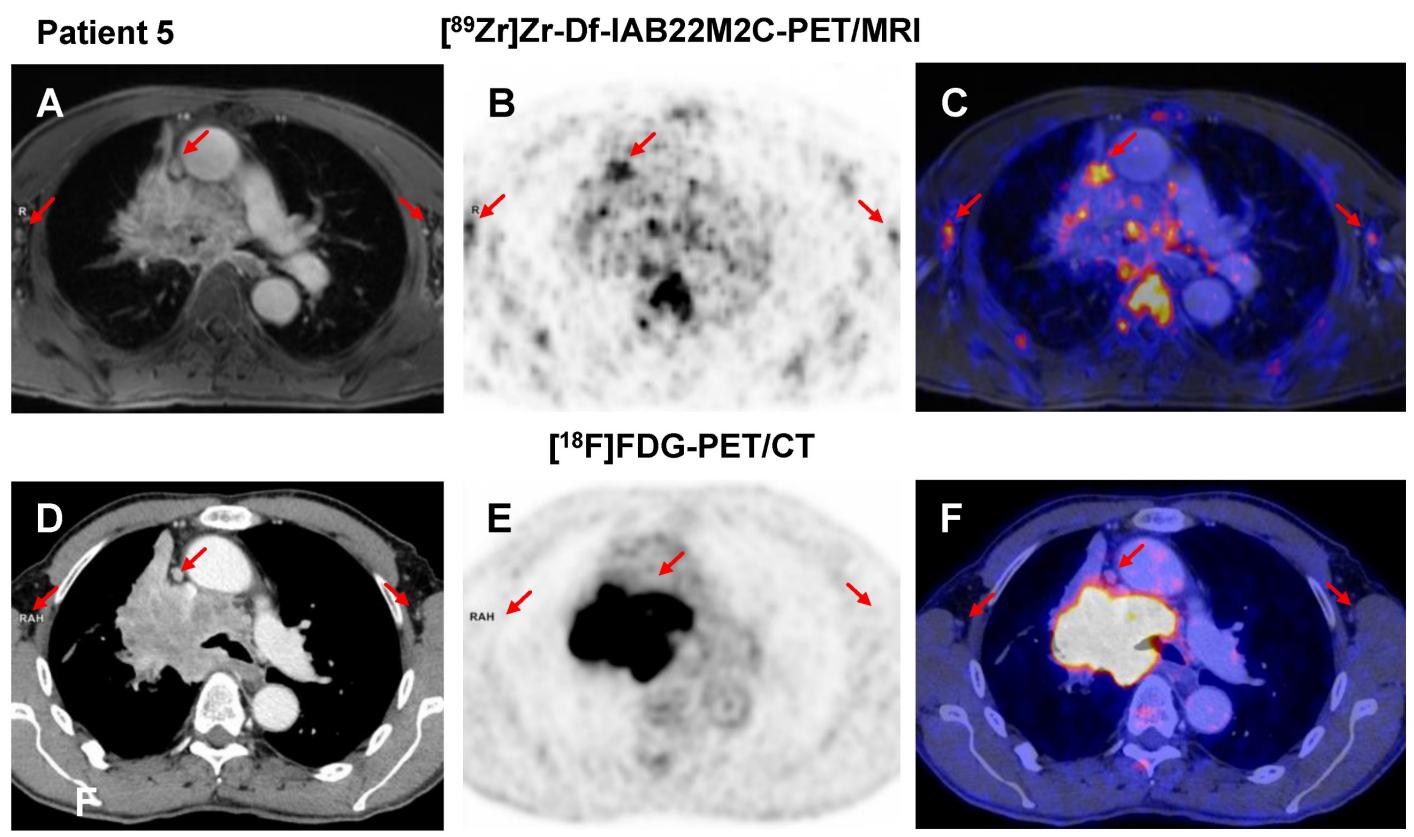
** A-C:** Patient 5 presented with moderate [^89^Zr]Zr-Df-IAB22M2C uptake in several mediastinal lymph nodes next to the primary tumor independent of any previous cancer treatment. **D-F:** In the previous [^18^F]FDG PET/CT scan, the mediastinal lymph nodes of Patient 5 were not considered suspicious for metastasis.

The comparison of [^89^Zr]Zr-Df-IAB22M2C and [^18^F]FDG PET in the NSCLC patient without ICT (Patient 5) revealed multiple mediastinal lymph nodes with moderate [^89^Zr]Zr-Df-IAB22M2C uptake next to the primary tumor (Figure 4A-C), which, however, were not considered suspicious in the previous [^18^F]FDG PET/CT scan (Figure 4D-F). This suggests a nonmetastatic inflammatory process as the main cause for the pronounced [^89^Zr]Zr-Df-IAB22M2C uptake in these lymph nodes.

In our cohort, the intense [^89^Zr]Zr-Df-IAB22M2C uptake of nonmetastatic lymph nodes was not associated with a therapeutic response, as we observed an identical [^89^Zr]Zr-Df-IAB22M2C uptake pattern in lymph nodes in both ICT-responsive and nonresponsive metastatic tumor patients (Figure 3A-B).

**Figure 5:**
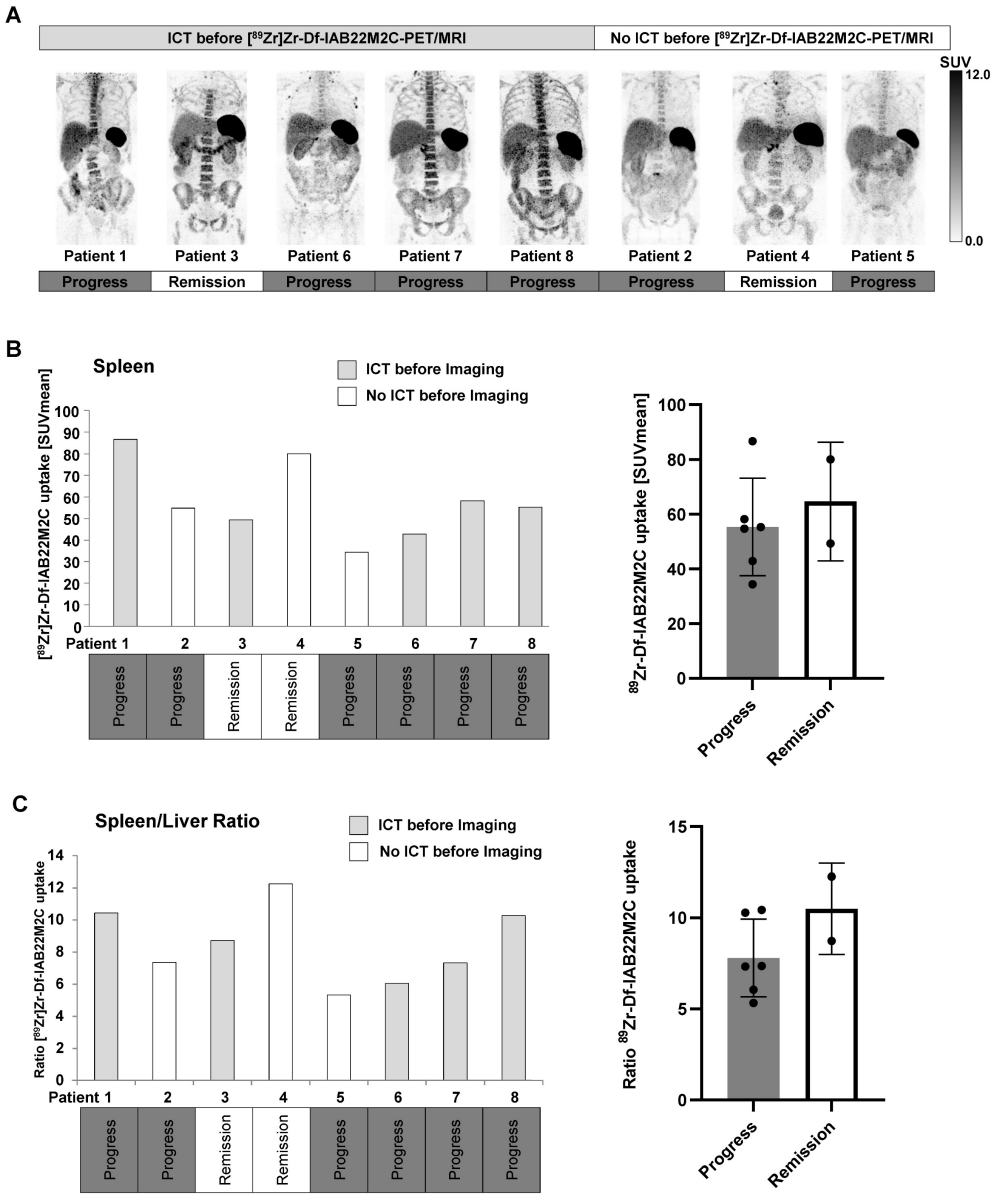
** A:** High [^89^Zr]Zr-Df-IAB22M2C uptake in the bone marrow was observed in the majority of the patients with ICT pretreatment, whereas patients without ICT pretreatment exhibited relatively low [^89^Zr]Zr-Df-IAB22M2C uptake in the bone marrow. **B:** A high variability of splenic [^89^Zr]Zr-Df-IAB22M2C uptake was determined in our patient cohort independent of ICT pretreatment or therapy response. **C:** The ratio of spleen to liver [^89^Zr]Zr-Df-IAB22M2C uptake was not directly correlated with previous ICT. Nevertheless, both patients with long-term remission (> one year remission under ICT; Patients 3 and 4) displayed a considerably high spleen-to-liver ratio.

Surprisingly, four out of five patients who underwent ICT before CD8 PET/MRI exhibited strongly pronounced [^89^Zr]Zr-Df-IAB22M2C uptake in the bone marrow (Figure 5A). Interestingly, we observed in patients without ICT pretreatment a generally lower [^89^Zr]Zr-Df-IAB22M2C uptake in the bone marrow when compared to the bone marrow of most of the ICT pretreated patients (Figure 5A and Table 1).

The spleen was the organ with the highest [^89^Zr]Zr-Df-IAB22M2C uptake in all patients regardless of the tumor entity and the patients' pretreatment. Moreover, splenic [^89^Zr]Zr-Df-IAB22M2C uptake displayed high variability in our patients without any correlation with ICT pretreatment or therapy response (Figure 5B).

Six out of eight patients exhibited progressive disease in the subsequent weeks and months after [^89^Zr]Zr-Df-IAB22M2C PET/MRI. Strikingly, both patients with long-term remission upon ICT (Patients 3 and 4; longer than one year of remission) displayed a noticeably elevated spleen to liver ratio (Figure 5C) and a relatively high [^89^Zr]Zr-Df-IAB22M2C uptake in the lymph nodes compared to the other patients (Figure 3A-B). Additionally, the spleen to blood pool ratio in ICT-responsive Patient 4 was among the highest within the cohort, while the lymph node to blood ratio was relatively high in ICT-responsive Patients 3 and 4 (Figure S3A-B). In contrast, the bone marrow to blood pool ratio was relatively low in the two ICT responders when compared to the other patients (Figure S3C). A lower spleen to liver ratio (Figure 5C) and lymph node uptake were associated with cancer progression in four out of six patients.

Thus, an elevated [^89^Zr]Zr-Df-IAB22M2C spleen to liver, spleen to blood, and lymph node to blood ratio, a low bone marrow to blood ratio, and a high [^89^Zr]Zr-Df-IAB22M2C lymph node uptake seem to be more associated with ICT responsiveness. In contrast, a low [^89^Zr]Zr-Df-IAB22M2C spleen to liver ratio and a low [^89^Zr]Zr-Df-IAB22M2C lymph node uptake might be associated with cancer progression.

**Figure 6:**
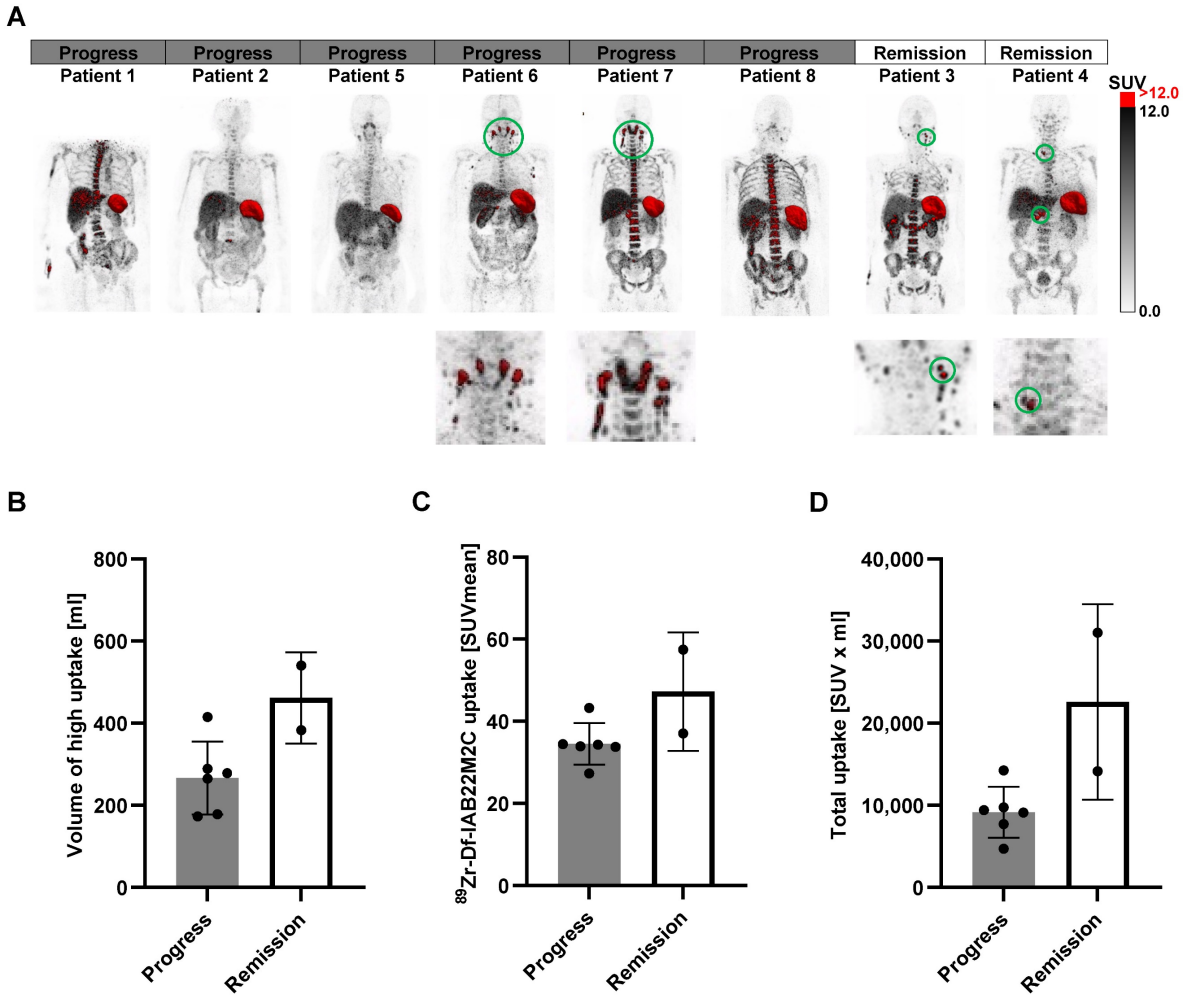
** A:** The areas with the highest [^89^Zr]Zr-Df-IAB22M2C uptake (SUV > 12) were segmented (red). **B:** The volume of the high [^89^Zr]Zr-Df-IAB22M2C uptake areas was enhanced in the two responders to ICT compared with most nonresponders. **C/D:** The mean SUVs of these high [^89^Zr]Zr-Df-IAB22M2C uptake areas and the total uptake showed a tendency toward elevated values in responders compared to nonresponders.

Next, we segmented the areas with the highest [^89^Zr]Zr-Df-IAB22M2C uptake (SUV > 12; CD8 high areas), as these regions represent tissues with a dense CD8^+^ T-cell infiltrate (Figure 6A). We have chosen SUV > 12 as a cutoff value as this excludes virtually all the uptake in the bone marrow, which besides the spleen is the organ of the predominant [^89^Zr]Zr-Df-IAB22M2C uptake in most of the patients. The volume of these areas was clearly higher in the two patients responding to ICT than in most of the nonresponders (Figure 6B). Additionally, the mean SUV in those areas (Figure 6C) and the total uptake (Figure 6D) revealed a tendency toward higher values in the ICT responders than in the nonresponders. Interestingly, the areas of high [^89^Zr]Zr-Df-IAB22M2C uptake included singular lymph nodes in the cervical and supraclavicular regions in both ICT responding patients (Figure 6A). The non-responders Patient 5 and 8 also displayed singular cervical lymph nodes with [^89^Zr]Zr-Df-IAB22M2C tracer uptake but the uptake of these lymph nodes did not reach the chosen threshold of SUV > 12 (Figure 6A).

In contrast, a generalized elevated uptake in multiple cervical lymph nodes and Waldeyer's tonsillar ring was present in two nonresponding patients and appeared not associated with an efficient antitumoral immune response (Figure 6A; Patients 6 and 7). Therefore, the differentiation of a high [^89^Zr]Zr-Df-IAB22M2C uptake in single lymph nodes from a generally elevated uptake in the cervical and supraclavicular lymph node region might be of special importance for the interpretation of the [^89^Zr]Zr-Df-IAB22M2C PET results. Finally, pretreatment with ICT did not result in significant differences in the [^89^Zr]Zr-Df-IAB22M2C uptake patterns in those areas (SUV > 12), although a higher variation in the determined values was observed in the patients without previous ICT (Figure S4A-D).

## Discussion

To date, cancer immunotherapies, such as ICT, are broadly available for multiple tumor entities. However, diagnostic capabilities to differentiate responders from nonresponders are still insufficient, and available imaging modalities are limited in predicting therapy response or irAEs. [^18^F]FDG PET/CT is widely used as a very sensitive method for the initial staging and monitoring of various cancer types as well as response assessment to therapy 51, 52. However, this method lacks specificity, especially with regard to its application to ICT. Reinfeld et al. recently revealed that a large proportion of glucose metabolism, as measured by [^18^F]FDG PET, is not exclusively related to viable tumor cells but also to activated immune cells 53.

Although the exact immunological processes in ICT-treated patients remain elusive, CD8^+^ T cells are critically involved in the ICT-induced antitumoral immune response 54. In recent years, several efficient immune cell tracking approaches have been developed, but the translation of many preclinical approaches into clinical application has faced challenges 55, 56. The radiolabeled antibody fragment (minibody) [^89^Zr]Zr-Df-IAB22M2C has been engineered to improve on the disadvantages often seen with full antibodies for *in vivo* imaging, in particular by reducing the long plasma half-life. To avoid interactions with Fc receptors and the associated immune activation, the Fc region was replaced by a pharmacologically inert domain.

Here, we present our first clinical experiences with [^89^Zr]Zr-Df-IAB22M2C PET/MRI in eight patients undergoing or eligible for ICT. To date, only limited data about the uptake pattern of the [^89^Zr]Zr-Df-IAB22M2C tracer, especially in lymphoid organs, have been available. Therefore, our observations in 8 tumor patients add additional information about the potential of [^89^Zr]Zr-Df-IAB22M2C to assess systemic immunological processes that are crucial for successful anticancer immune responses via the visualization of CD8^+^ T-cell biodistribution, migration, and homing patterns. Within our small retrospective patient cohort, we detected elevated CD8-dependent [^89^Zr]Zr-Df-IAB22M2C uptake in two metastases of two patients. Unfortunately, the two [^89^Zr]Zr-Df-IAB22M2C accumulating lesions were not eligible for resection. Thus, we were unable to cross validate the elevated [^89^Zr]Zr-Df-IAB22M2C tracer uptake by CD8 immunohistochemistry. Nevertheless, two of the metastases without enhanced [^89^Zr]Zr-Df-IAB22M2C uptake were resected: the pararenal metastasis of Patient 1 and the brain metastasis of Patient 3. CD8 immunohistochemistry of the pararenal metastasis revealed a very faint CD8^+^ T-cell infiltrate in the center of the metastasis, thus cross-validating the lack of [^89^Zr]Zr-Df-IAB22M2C uptake. Strikingly, some areas with dense infiltration of CD8^+^ T cells were found at the margins of the metastasis. The immunohistochemistry staining for CD8 in Figure 1F demonstrates that the dense infiltration of CD8^+^ T cells is located in a very thin layer at the margin of the lesion. As this layer is only a few micrometers thick and only a few CD8^+^ T cells were located in the tumor center, the partial volume effect could explain why this infiltration of CD8^+^ T cells at the margin was not reflected by the [^89^Zr]Zr-Df-IAB22M2C PET images.

In the case of the brain metastasis of Patient 3, the breakdown of the blood brain barrier in melanoma metastasis should enable a sufficient delivery of the tracer into the malignant tissue. Since there are only very few CD8^+^ T cells located in this metastasis according to immunohistochemistry (Figure S1), no relevant uptake was observed in the [^89^Zr]Zr-Df-IAB22M2C PET.

In recent years, it has become evident that the amount and localization of T-cell infiltration in the tumor is a major predictor of patient outcome 57-59. The distinction of a) immunological “hot” tumors with dense immune infiltrates, b) immune “deserts” without relevant immune infiltrate and c) immune excluded tumors with an immune infiltrate at the tumor margins has been proposed by different authors 60, 61. The CD8 immunohistochemistry of the pararenal metastasis of Patient 1 correlated very well with the noninvasive CD8 PET/MR imaging and would classify the lesion into the so-called immune “excluded” phenotype. Nevertheless, larger-scale prospective trials with histological cross-validation are needed to validate whether [^89^Zr]Zr-Df-IAB22M2C PET is applicable to distinguish immunological “hot” tumors with a dense immune infiltrate from immune-excluded or immune-deserted tumors.

In addition, it must be considered that the presence of CD8^+^ T cells does not guarantee that they are functional cells. Consequently, additional characterization of the tumor microenvironment, such as metabolic parameters (glucose metabolism and lactate production), or acidity (pH) might be necessary to further evaluate the effectiveness of the T-cell infiltrate in regard to an efficient antitumor immune response.

In our small cohort of metastatic patients, [^89^Zr]Zr-Df-IAB22M2C uptake in the metastatic lesions was unable to predict successful ICT. Therefore, we focused on primary and secondary lymphatic organs, as they are essential hubs of the cancer immune response.

During ICT, the primary and secondary lymphatic organs are considered to be highly involved in the elicited systemic immune response in cancer patients.

Recently, our group performed preclinical translational as well as prospective clinical studies with [^18^F]FDG PET for the assessment of metabolic changes in primary and secondary lymphoid organs before and after the onset of ICT to visualize and quantify the systemic response 51, 62. These studies showed that elevated splenic glucose metabolism was related to responsiveness to ICT 62 and may serve as an early treatment response marker 51. In addition, we observed that glucose metabolism in the bone marrow, a primary lymphatic organ, was increased in patients responsive to ICT. Interestingly, elevated glucose metabolism in the bone marrow, even before the start of ICT, was able to predict treatment response 51, 62. Nevertheless, it must be considered that the increased but probably relatively nonspecific glucose metabolism in the primary and secondary lymphatic organs might be a feature that can also be found in a variety of both physiological and pathological conditions.

In our patient cohort, a high interpatient [^89^Zr]Zr-Df-IAB22M2C uptake variability within the primary and secondary lymphoid organs was observed. Nonmalignant lymph nodes with high [^89^Zr]Zr-Df-IAB22M2C uptake were found in the majority of the patients, especially in the cervical and thoracic regions. Interestingly, the presence or absence of ICT treatment did not impact the elevated [^89^Zr]Zr-Df-IAB22M2C uptake in these lymph nodes. Furthermore, increased [^89^Zr]Zr-Df-IAB22M2C uptake in representative lymph nodes was associated with a significant decrease in ADC values assessed by DWI (Figure 3C). An increase in cellularity is inversely correlated with the diffusion assessed by ADC values in MRI 63; therefore, the observed decrease in diffusion in the lymph nodes with increased [^89^Zr]Zr-Df-IAB22M2C uptake might be in line with cellular proliferation in activated lymph nodes, which is accompanied by enhanced CD8^+^ T-cell infiltration.

Generally, CD8^+^ T cells are critically involved in adaptive immune responses, including the elimination of malignant cells but are also involved in other functions, such as the removal of virus-infected cells 17. Therefore, many physiological and pathological processes within the primary and secondary lymphatic organs might influence the [^89^Zr]Zr-Df-IAB22M2C biodistribution. Thus, patient-specific physiological (aged immune system) and pathological processes (viral infections) might interfere with the holistic [^89^Zr]Zr-Df-IAB22M2C PET signature.

Interestingly, 4 out of 6 patients with tumor progression after ICT presented with a lower spleen-to-liver ratio in comparison to the two ICT-responsive patients who were in remission. These findings are in line with preclinical data, where ICT-induced glucose metabolism of the spleen was associated with an elevated number of neutrophils and a reduced number of infiltrating T cells 62. In accordance with this, our prospective study mentioned above revealed elevated [^18^F]FDG uptake in the spleen of most ICT-responsive patients, while nonresponsive patients did not exhibit any significant differences. Moreover, multiple reasons for dysfunctional CD8^+^ T cells within the tumor microenvironment have been discovered, which are major hurdles for ICT efficacy 13. [^89^Zr]Zr-Df-IAB22M2C PET alone might not be suitable to evaluate the functionality of CD8^+^ T cells as it does not allow a differentiation of CD8^+^ T-cell subsets with distinct functionality like e.g. exhausted or effector CD8^+^ T cells. In recent years, many of markers have been identified, but nevertheless a single predictive biomarker for successful ICT is still missing.

Thus, dual tracer approaches might be required for the identification of efficient CD8^+^ T-cell function and potentially enable the detection of therapy response. As a second tracer in addition to [^89^Zr]Zr-Df-IAB22M2C, newly developed tracers, such as [^18^F]-arabinosyl guanine 64 and the granzyme B targeting tracer [^68^Ga]-NOTA-GZP 65, might provide additional information on T-cell functionality. Additionally, the recently developed PD-1 and PD-L1 PET ligands could offer enormous potential in combination with CD8 imaging 66. Thus, PD-1 expression patterns within tissues with a pronounced CD8^+^ T-cell infiltrate might allow the differentiation of progenitor and terminal exhausted CD8^+^ T-cells.

However, conventional [^18^F]FDG could also potentially provide evidence of T-cell activation, as we were able to demonstrate previously that the distribution of [^18^F]FDG in primary and secondary lymphoid organs is accompanied by successful cancer immune responses 62.

Additionally, the highly dynamic temporal changes in the tumor immune infiltrate as well as in the lymphoid organs must be considered when analyzing the CD8^+^ T cell distribution. Of note, the timing of the [^89^Zr]Zr-Df-IAB22M2C PET investigations could not be controlled in this retrospective setting. To our knowledge, it is currently unclear to which extent this influences the predictive power of [^89^Zr]Zr-Df-IAB22M2C PET and how large the pathophysiological differences between individual patients with distinct pretreatments, tumor entities, localization of primary tumors and metastases are.

Nevertheless, in this rather small clinical dataset we could not observe obvious differences between ICT naïve patients and patients on ICT. Additionally, factors like the tumor mass, the localization of the tumors and metastasis might influence the CD8^+^ T cell distribution. In addition, age, tumor entity and comorbidities are confounding factors which could not be considered here.

All these influences need to be explored in large scale prospective trials to determine the ideal time point and conditions to assess CD8^+^ T cell distribution patterns that are predictive for successful ICT.

The segmentation of the areas with the highest [^89^Zr]Zr-Df-IAB22M2C uptake (SUV > 12) revealed a higher volume in the two patients responding to ICT than in most of the nonresponders (Figure 6B), including singular lymph nodes in the cervical and supraclavicular regions (Figure 6A). This is of particular interest, as the “stem-like” progenitor exhausted T cells in the tumor draining lymph node tissue are considered crucial for successful PD-1 therapy 48.

In contrast, the notable generalized and enhanced [^89^Zr]Zr-Df-IAB22M2C uptake in multiple cervical lymph nodes and Waldeyer's tonsillar ring in Patients 6 and 7 (Figure 6A) appears to be independent of an efficient antitumor response and is most likely due to nonspecific inflammatory processes such as respiratory infections.

If cases with generalized lymphadenopathy are evaluated with the utmost caution, the analysis of the areas with high [^89^Zr]Zr-Df-IAB22M2C uptake by whole-body PET/MRI might be a way to identify the lymphoid tissue, which is crucial for an efficient anticancer immune response. In line with this observation, the analysis of the [^89^Zr]Zr-Df-IAB22M2C uptake in lymph nodes (Figure 3C) revealed a tendency toward higher uptake values in the patients responding to ICT.

Previously, the CD8 expression level on T-cells has been correlated with T-cell reactivity 67, 68, but the significance of these published results in regard to an effective antitumoral immune response induced by progenitor exhausted CD8^+^ T-cell via PD-1 blockade, is largely unclear. Nevertheless, the lower ADC values determined by MRI within lymph nodes suggested that a higher cellular density due to the proliferation of CD8^+^ T-cells leads to higher CD8 expression. As mentioned above, progenitor exhausted T cells in the lymphoid tissue with the ability to proliferate are considered crucial for successful PD-1 therapy 48. Therefore, the combination of [^89^Zr]Zr-Df-IAB22M2C PET and ADC assessed by MRI could be a major advantage in characterizing the lymphatic tissue to identify responders to ICT.

Of note, subsequent prospective clinical studies are needed to explore how reliable these patterns are and whether they allow treatment stratification to improve the therapeutic outcome of patients with metastasized cancer. Because in all patients individual clinical decisions led to the acquisition of a [^89^Zr]Zr-Df-IAB22M2C PET/MRI, the investigated patients are mostly cases where clinicians were in doubt about the start of an ICT. This might explain the disproportional fraction of non-responders in this dataset. To investigate the findings of this retrospective study thoroughly we recently initiated a prospective study on patients with metastatic melanoma under ICT (EudraCT-number 2021-004328-13).

Nevertheless, our retrospective analysis of eight tumor patients revealed a large variability in [^89^Zr]Zr-Df-IAB22M2C tracer uptake, especially in primary and secondary lymphoid organs. In addition, several studies on tumor immunology demonstrated that the presence of CD8^+^ T cells in the tumor microenvironment alone is not sufficient to elicit an effective immune response against tumors 69. Consequently, better knowledge about the uptake characteristics of the [^89^Zr]Zr-Df-IAB22M2C tracer in primary and secondary lymphoid organs may thus be of importance when designing future prospective clinical studies that focus on the recognition of a systemic immune response. This should include thorough statistical planning and a clear definition of useful measurement parameters as well as an in-depth characterization of the assessed CD8^+^ T-cell subsets.

## Conclusion

Our first clinical data revealed the utility of [^89^Zr]Zr-Df-IAB22M2C PET/MRI to assess potential immune-related changes in CD8 expression within metastases and within primary and secondary lymphatic organs, which has not been reported thus far. The results suggest that the distribution of CD8^+^ T cells in the lymphatic organs as assessed by [^89^Zr]Zr-Df-IAB22M2C PET might be associated with the systemic immune response induced by ICT. Further prospective clinical trials are needed to gain deeper insights into the relationship of the detected CD8^+^ T-cell distribution and response to ICT.

## Supplementary Material

Supplementary figures and tables.

## Figures and Tables

**Table 1:** Patient characteristics.

Patient	1	2	3	4	5	6	7	8
Cancer type	Cutaneous melanoma	Uveal melanoma	Cutaneous melanoma	Cutaneous melanoma	Bronchial cancer	Cutaneous melanoma	Cutaneous melanoma	Sarcoma
Therapy before scan	Ipilimumab	Surgery	Nivolumab	Surgery	None	Pembrolizumab	Ipilimumab/Nivolumab	Nivolumab
Therapy after scan	Resection/Nivolumab	Ipilimumab/ Nivolumab	Resection/Nivolumab	Nivolumab	RCTx/Durvalumab	None	Nivolumab	Nivolumab
Outcome	Progress	Progress	Remission	Remission	Progress	Progress	Progress	Progress
Liver (SUVmean)	8.3	7.4	5.7	6.5	6.5	7.1	7.9	5.4
Spleen (SUVmean)	86.7	54.7	49.3	80.0	34.4	42.9	58.2	55.3
LN (SUVmean)	2.9	2.5	13.5	18.8	3.7	19.3	11.6	7.0
LN with highest uptake	Retroperitoneal	cervical left	cervical left	liver hilum	axillar left	cervical left	cervical left	submandibular left
Lumbar vertebra(SUVmean)	10.6	4.7	10.1	6.9	5.5	7.4	10.8	12.3
Blood pool (SUVmean)	1.6	1.4	1.7	1.5	1.7	1.5	1.2	1.5

RCTx (radiochemotherapy); LN (lymph node)
