# Supplementary material for: In vivo imaging of CD8+ T cells in metastatic cancer patients: first clinical experience with simultaneous [89Zr]Zr-Df-IAB22M2C PET/MRI
Source: Theranostics. 2023 Apr 17;13(8):2408–23. doi: 10.7150/thno.79976 (PMC10196830; doi:10.7150/thno.79976)
Supplement: Supplementary file 1 — Supplementary figures and tables. [file thnov13p2408s1.pdf]

Figure S1

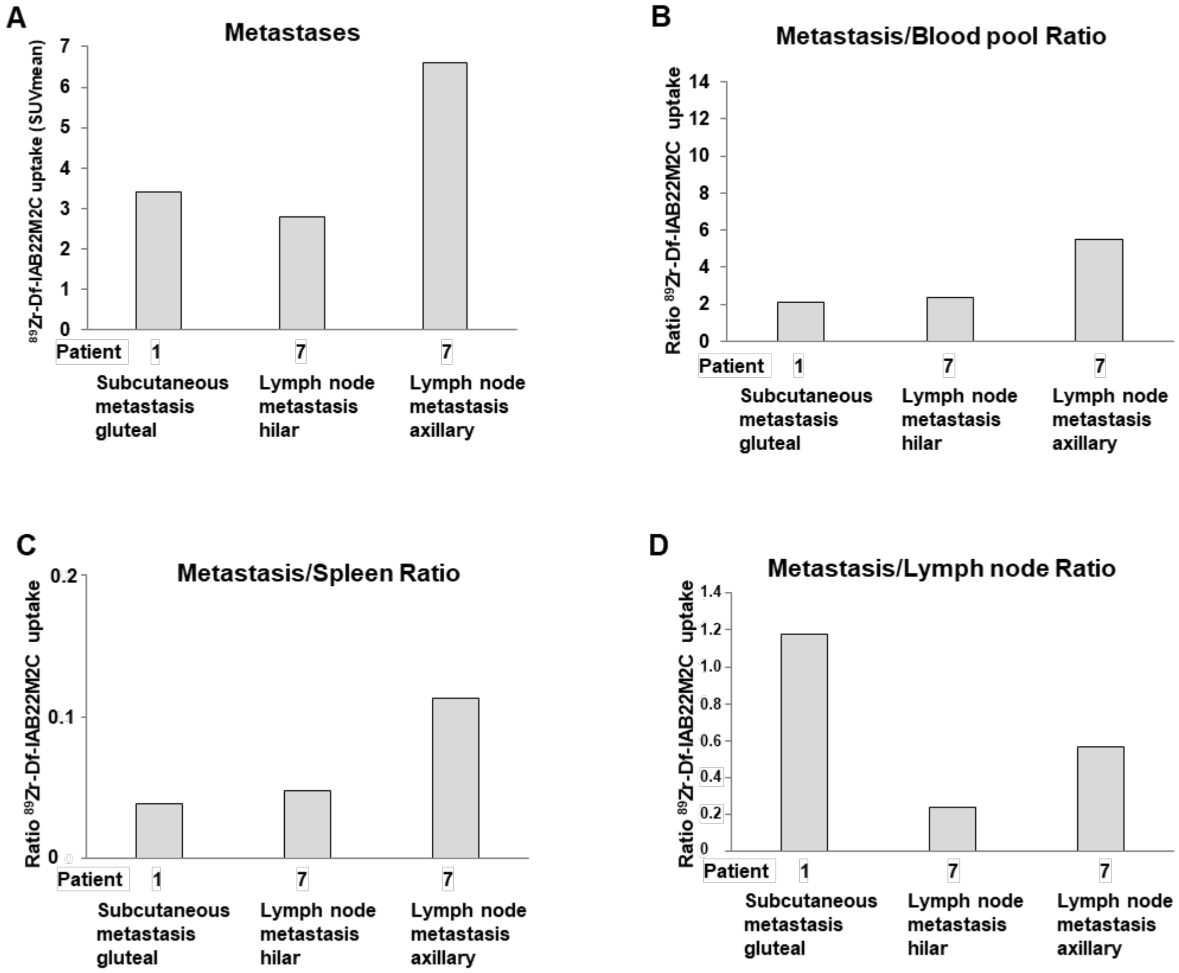

A: Quantification of the [^{89}Zr]Zr-Df-IAB22M2C uptake in the three metastases (subcutaneous; suspicious hilar and axillary lymph node) with increased uptake compared to the background **B-D:** Graphical representation of the metastasis (subcutaneous; suspicious hilar and axillary lymph node with the highest uptake) to blood pool [^{89}Zr]Zr-Df-IAB22M2C uptake ratios, the metastasis to spleen ratios and the metastasis to lymph node ratios.

Figure S2

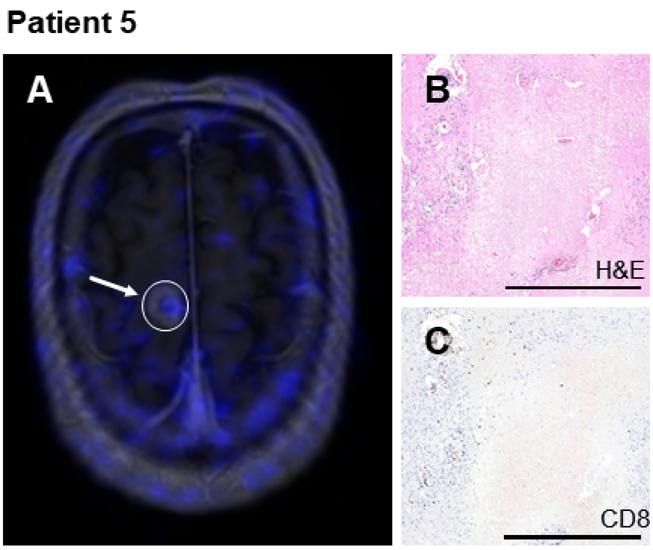

A: In the brain metastasis of Patient 3, no relevant [^{89}Zr]Zr-Df-IAB22M2C uptake was detectable. **B:** H&E histology of the brain metastasis six weeks after PET/MRI revealed necrotic tissue without vital malignant cells as well as white and gray matter interspersed with gemistocytic astrocytes, which is a typical finding postradiotherapy. **C:** In addition to some CD8$^+$ T cells perivascularly near the necrotic areas, only very few CD8$^+$ T cells were found in the brain parenchyma and necrotic areas.

Figure S3

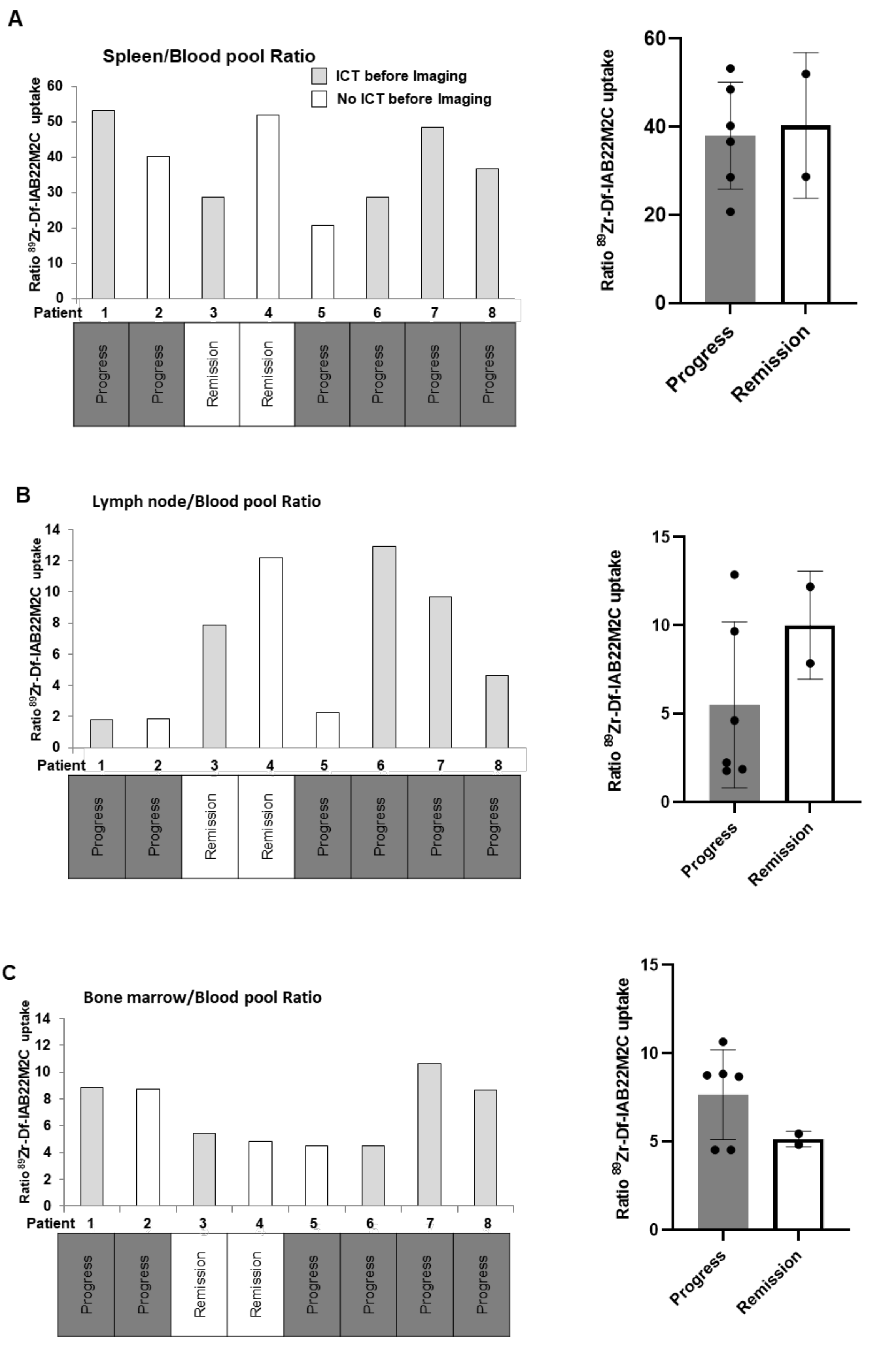
A
Spleen/Blood pool Ratio
ICT before Imaging
No ICT before Imaging
Ratio ^{89}Zr-Df-IAB22M2C uptake
Patient
Progress
Remission
B
Lymph node/Blood pool Ratio
C
Bone marrow/Blood pool Ratio

A: Calculation of the ratio of splenic [^{89}Zr]Zr-Df-IAB22M2C uptake to the blood pool. **B-C:** Uptake ratio of the lymph node with the highest uptake and the blood pool as well as uptake ratio of the area with the highest uptake in the bone marrow and the blood pool.

Figure S4

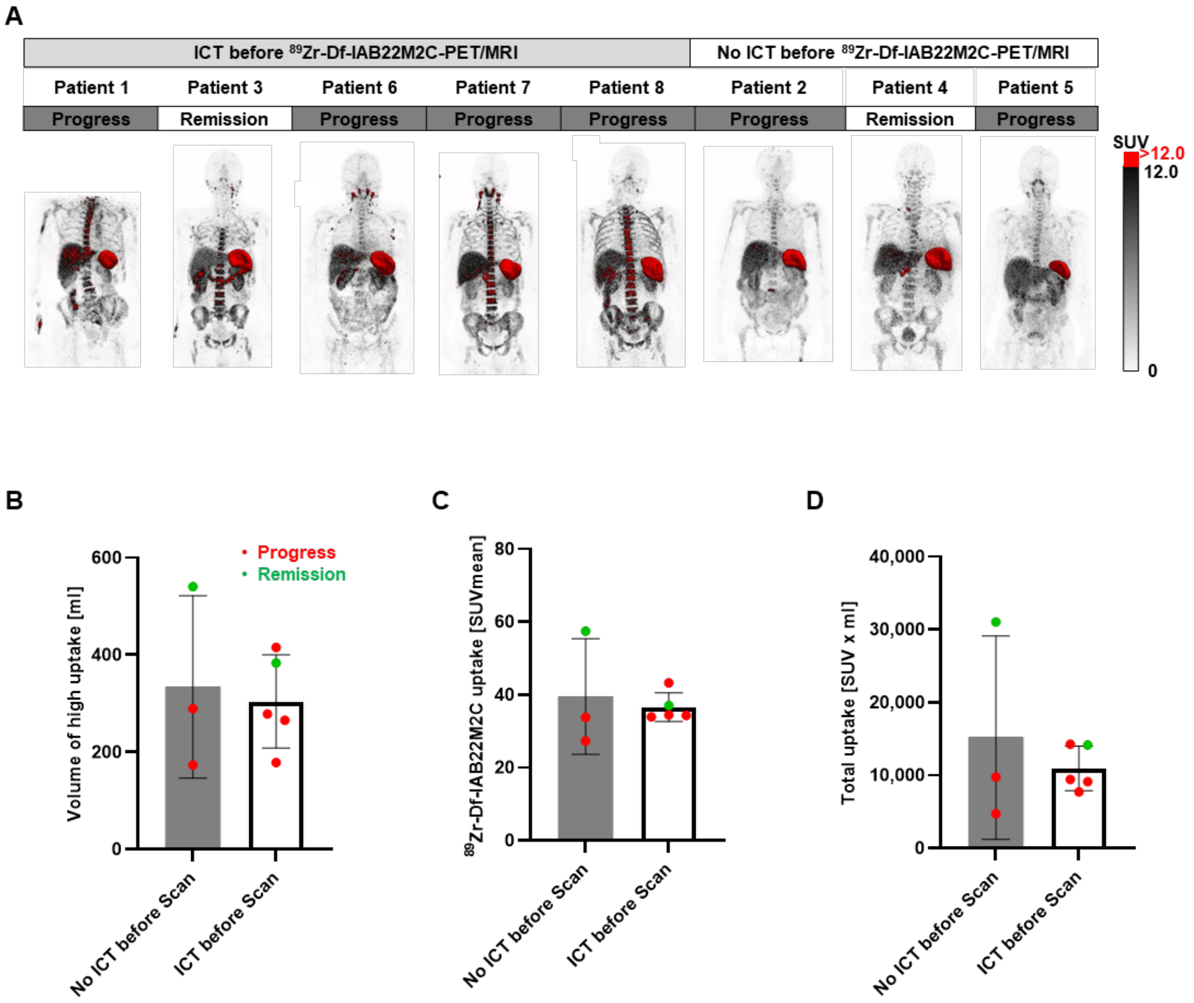

A: The areas with the highest [^{89}Zr]Zr-Df-IAB22M2C uptake (SUV >12) were segmented (red) and grouped according to the pretreatment. **B-D:** Pretreatment with ICT did not result in significant differences in volume, SUVmean or total uptake of [^{89}Zr]Zr-Df-IAB22M2C in those areas (SUV > 12), although a higher variation in the determined values was observed in the patients without previous ICT.

Table S1

Patient	Dose [MBq]	Effective radiation dose [mSv] according to Farwell et al. 2022
1	64.2	41.7
2	103.4	67.2
3	97.1	63.1
4	90.0	58.5
5	56.9	37.0
6	62.0	40.3
7	62.2	40.4
8	58.0	37.7

Table S2

Nr.	Days between previous [^{18}F]-FDG and [^{89}Zr]Zr-Df-IAB22M2C PET scans	Days between subsequent [^{18}F]-FDG and [^{89}Zr]Zr-Df-IAB22M2C PET scans
1	784	
2		88
3		426
4		
5	3	
6	533	122
7	34	63
8		
